# A synergistic approach for modulating the tumor microenvironment to enhance nano-immunotherapy in sarcomas

**DOI:** 10.1016/j.neo.2024.100990

**Published:** 2024-03-22

**Authors:** Fotios Mpekris, Myrofora Panagi, Antonia Charalambous, Chrysovalantis Voutouri, Christina Michael, Antonia Papoui, Triantafyllos Stylianopoulos

**Affiliations:** Cancer Biophysics Laboratory, Department of Mechanical and Manufacturing Engineering, University of Cyprus, Cyprus

**Keywords:** Mechanotherapeutics, Sonopermeation, Nanomedicine, Immune checkpoint inhibition, Ultrasound

## Abstract

The lack of properly perfused blood vessels within tumors can significantly hinder the distribution of drugs, leading to reduced treatment effectiveness and having a negative impact on the quality of life of patients with cancer. This problem is particularly pronounced in desmoplastic cancers, where interactions between cancer cells, stromal cells, and the fibrotic matrix lead to tumor stiffness and the compression of most blood vessels within the tumor. To address this issue, two mechanotherapy approaches–mechanotherapeutics and ultrasound sonopermeation–have been employed separately to treat vascular abnormalities in tumors and have reached clinical trials. Here, we performed *in vivo* studies in sarcomas, to explore the conditions under which these two mechanotherapy strategies could be optimally combined to enhance perfusion and the efficacy of nano-immunotherapy. Our findings demonstrate that combination of the anti-histamine drug ketotifen, as a mechanotherapeutic, and sonopermeation effectively alleviates mechanical forces by decreasing 50 % collagen and hyaluronan levels and thus, reshaping the tumor microenvironment. Furthermore, the combined therapy normalizes the tumor vasculature by increasing two-fold the pericytes coverage. This combination not only improves six times tumor perfusion but also enhances drug delivery. As a result, blood vessel functionality is enhanced, leading to increased infiltration by 40 % of immune cells (CD4^+^ and CD8^+^ T-cells) and improving the antitumor efficacy of Doxil nanomedicine and anti-PD-1 immunotherapy. In conclusion, our research underscores the unique and synergistic potential of combining mechanotherapeutics and sonopermeation. Both approaches are undergoing clinical trials to enhance cancer therapy and have the potential to significantly improve nano-immunotherapy in sarcomas.

## Highlights

• Abnormalities in the tumor microenvironment pose major barriers to sarcoma therapy.
• Mechanotherapy and sonopermeation synergistically overcome these abnormalities.
• Combined treatment increases drug delivery and immune cells infiltration.
• Proposed strategy enhances anti-tumor immunity and nano-immunotherapy efficacy.

## Graphical abstract

**Figure:**
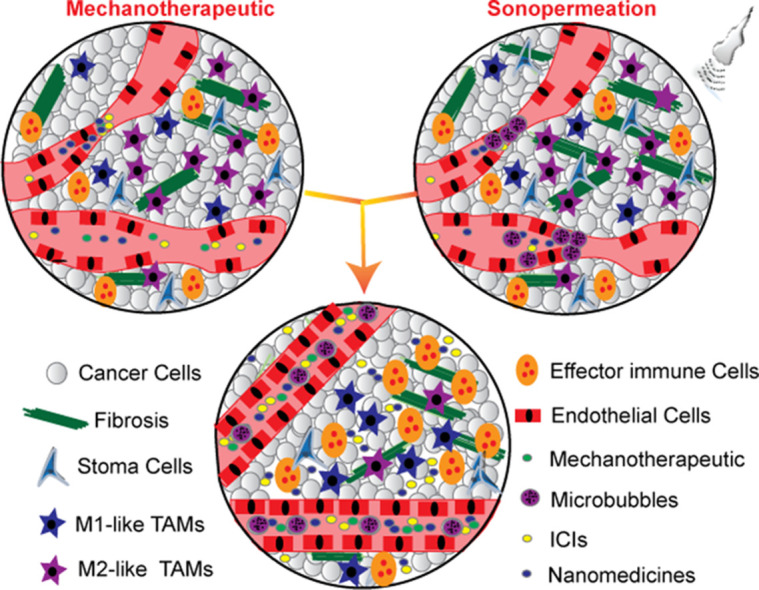

## Introduction

Inefficient delivery of cellular and molecular medicines to solid tumors can reduce dramatically the efficacy of treatment and thus, affect negatively the quality of life and survival of cancer patients [1]. In highly desmoplastic cancers, such as types of sarcomas, interactions among cancer cells, stromal cells, and the fibrotic extracellular matrix (ECM) (i.e., excess deposition of collagen and hyaluronan) result in tumor stiffening and accumulation of intratumoral mechanical forces that are exerted on tumor blood vessels, causing vessel compression [2], [3], [4], [5], [6]. This in turn leads to reduced tumor blood flow and drug delivery, rendering tumors hypo-perfused and hypoxic [3,[7], [8], [9]]. Hypo-perfusion not only reduces drastically drug delivery, but along with hypoxia helps cancer cells evade the immune system and increases their invasive and metastatic potential [10], [11], [12], [13], [14], [15]. Particularly, hypo-perfusion hinders immune cells infiltration into the tumor, while hypoxia renders tumor microenvironment (TME) immunosuppressive, reprograming tumor-associated macrophages (TAMs) from an immunosupportive M1 type toward an immunosuppressive M2 type and attenuates the killing potential of effector immune cells [16], [17], [18], [19], [20], [21].

A therapeutic strategy to decompress vessels and improve perfusion is the use of mechanotherapeutics to alleviate stiffness and mechanical forces in tumors [22]. Mechanotherapeutics are often common drugs (e.g., antihypertensive, anti-fibrotic, antihistamine) that are repurposed to modulate the TME by targeting ECM components or reprogramming Cancer Associated Fibroblasts (CAFs) to re-open compressed vessels and improve perfusion and delivery of drugs [8,19,[23], [24], [25], [26], [27], [28], [29]]. The anti-hypertensive losartan was the first drug that was tested in a phase II clinical trial, and it was found that its combined use with FOLFIRINOX in pancreatic cancer patients, increased dramatically the number of patients eligible for tumor surgical resection [30]. Importantly, another phase II clinical trial to test the use of losartan and chemoradiation in combination with immune checkpoint inhibition in pancreatic cancer patients has been initiated (clinical trials.gov identifier NCT03563248). Furthermore, in a previous study we employed ketotifen, a second-generation noncompetitive H1-antihistamine and mast cell stabilizer, approved worldwide as a common antihistamine drug. We demonstrated that ketotifen can have a dual role by acting as a mechano- and immuno-modulator of the TME in sarcomas [31]. Based on these studies, we initiated a phase II trial to test the potential of ketotifen to improve chemotherapy in patients with sarcomas (EU clinical trials register, EudraCT No: 2022-002311-39). However, mechanotherapeutics cannot decompress the majority of the vessels but only a percentage of them [26].

Ultrasound in medical diagnostics is a safe and widely applied real-time imaging modality. During the last decades, it has also been increasingly studied for therapeutic purposes [32,33]. The use of ultrasound in the presence of exogenous gas bubbles (i.e., microbubbles) can lead to the development of local forces strong enough to cause membrane permeabilization of cells. Use of ultrasound and microbubbles, known as sonopermeation, have demonstrated improved effects on conventional chemotherapeutics in pancreatic ductal adenocarcinoma cancer (PDAC) and glioblastoma patients [34], [35], [36]. Furthermore, when exposed to ultrasound and microbubbles, increased tumor uptake of nanoparticle liposomal doxorubicin (Doxil) has been demonstrated in subcutaneously colorectal [37] and prostate adenocarcinoma [38] murine models and enhanced encapsulation of cabazitaxel in prostate cancer [39]. It has been also shown to increase infiltration of cytotoxic T-cells in colorectal cancer and melanoma [40,41] and NK cells in ovarian cancer [42] murine models. A clinical investigation using sonopermeation to treat solid tumors evaluated the safety and potential toxicity of combining gemcitabine with microbubbles under sonication in ten inoperable pancreatic cancer patients [34]. The study concluded that sonopermeation and chemotherapy resulted in no additional toxicities. Furthermore, the treatment enhanced the clinical efficacy of gemcitabine and extended survival in patients with pancreatic cancer. Several similar studies have been initiated in patients suffering from breast cancer, liver metastasis resulting from primary colon cancer, and pancreatic cancer [35,[43], [44], [45]] (NCT03322813, NCT04146441, NCT03458975, NCT03477019, NCT04021420 and NCT03385200). However, hypo-perfusion due to vessel compression compromises efficacy of sonopermeation as the microbubbles cannot effectively and uniformly be delivered to the entire tumor, leading to local effects and compromised efficacy of this therapeutic strategy [46,47].

Therefore, mechanotherapeutics can open up some tumor vessels and improve tumor perfusion to significantly increase the distribution of microbubbles in the tumor and thus, enhance the efficacy of sonopermeation. Furthermore, it has been recently reported that sonopermeation can reduce intratumoral solid stress and thus, improve perfusion [48], but the underlying mechanisms are still unknown. Therefore, sonopermeation could support mechanotherapeutics use to further alleviate mechanical forces in tumors and potentially opening up more tumor vessels, allowing more microbubbles and anti-cancer drugs entering the tumor and thus, creating a positive feedback loop. Here, using two murine sarcoma models, we identified using a clinical ultrasound device, the optimal values of mechanical index and number of cycles of sonopermeation that can improve tumor perfusion and drug delivery. Additionally, we demonstrated that combination of ketotifen and sonopermeation can optimally induce stroma and vascular normalization by targeting extracellular matrix and vessel components, alleviate mechanical forces and enhance treatment efficacy of nano-immunotherapy.

## Materials and methods

A detailed description of methods used for fluorescence immunohistochemistry, flow cytometry and drug delivery studies are presented in the Supplementary Material.

### Cell culture

MCA205 fibrosarcoma cells (SCC173, Millipore) were cultured in RPMI-1640 (LM-R1637, biosera) containing 2 mM L-glutamine (TMS-002-C, Sigma-Aldrich), 1 mM sodium pyruvate (TMS-005-C, Sigma-Aldrich), 10 % fetal bovine serum (FBS, FB-1001H, biosera), 1x non-essential amino acids (TMS-001-C, Sigma-Aldrich), 1 % antibiotics (A5955, Sigma) and 1x β-mercaptoethanol (ES-007-E, Sigma). K7M2-WT osteosarcoma cells (CRL2836, ATCC) were cultured in DMEM (LM-S2041, biosera) containing with 10 % FBS and 1 % antibiotics. Cells were incubated at 37 ºC/ 5 % CO_2_.

### Drugs and reagents

Ketotifen fumarate salt (K2628, Sigma) was dissolved in sterilized normal saline (9 % NaCl in ddH_2_O, w/v). Doxil (Pegylated liposomal doxorubicin, Janssen Pharmaceuticals) was purchased as already made solution (2 mg/ml). The immune checkpoint inhibitor (ICI) mouse monoclonal anti-PD-1 antibody (CD279, clone RMP1-14) was purchased from BioXCell and diluted in InVivoPure pH 7.0 Dilution Buffer.

### Ultrasound setup for sonopermeation

A clinical diagnostic ultrasound scanner (Philips EPIQ Elite) in combination with a C5-1 curvilinear probe (Philips) was used to apply the therapeutic ultrasound sonopermeation method **(Supplementary Fig. 1)**. Sonopermeation therapy was done in Pulsed Wave (PW) Doppler mode. The transmission frequency of the transducer is established by the system and it is unalterable. The acoustic pressure was varied by changing the mechanical index (MI). The number of cycles per pulse (NoC) was varied by changing the sample volume (SV) as described previously [49], while the MI was kept constant. The values that were used for Mechanical Index were: Low MI=0.2, Medium MI=0.6 and High MI=1.3, and for Number of cycles per pulse were: Low NoC=16 (SV=5mm), Medium NoC=32 (SV=10mm) and High NoC=64 (SV=20mm) [50], [51], [52], [53], [54]. Clinically approved ultrasound contrast agent (SonoVue®) was used as the microbubble for sonopermeation. The *in-vivo* life time of microbubbles in mice is 4–5 min [34], thus we chose to inject intravenously (i.v.) 2 boluses with a 2 min break between to ensure microbubbles were present continuously throughout the whole treatment. The duration of each injection was 30 s. Mice were subjected to sonopermeation for a total time of 5 min.

### Syngeneic tumor models and treatment protocols

Animal models: Fibrosarcoma tumors were generated by inoculating 6‐8 week old C57BL/6 female and male (equal number) mice with 2.5 × 10^5^ MCA205 cells in 50 µL of serum‐free medium into the leg muscle. Osteosarcoma tumors were generating by implanting to the tibia 1 mm^3^ dissected tumor chunks from K7M2-WT tumors into 6‐8 week old BALB/c female and male (equal number) mice. All experiments were conducted in accordance with the animal welfare regulations and guidelines of the European Union (European Directive 2010/63/EE and Cyprus Legislation for the protection and welfare of animals, Laws 1994–2013) under a license acquired and approved (CY/EXP/PR.L14/2019, CY/EXP/PR.L15/2019, CY/EXP/PR.L03/2020) by the Cyprus Veterinary Services committee, the Cyprus national authority for monitoring the welfare of animals in research. Animals were anesthetized by intraperitoneal (i.p.) injection of Avertin (200mg/kg).

Sonopermeation response study: When tumors reached an average volume of 300 mm^3^, mice were randomized into six groups (n=4 male and 4 female per group) as presented in **Supplementary Table 1**. Two hours prior sonopermeation and one hour after sonopermeation, we performed shear wave elastography (SWE) and contrast enhanced ultrasound (CEUS) (described below) to quantify tumor stiffness and perfusion.

Antitumor activity of nano-immunotherapy in murine sarcoma models: When tumors reached an average size of 100 mm^3^, mice were randomized in the following groups (n=8-10 per group): Control group, ketotifen (10 mg/kg, i.p.), sonopermeation, ketotifen+sonopermeation, Doxil (3mg/kg, i.v.)+immune checkpoint inhibitor-ICI (anti-PD-1, 10 mg/kg, i.p.), ketotifen+Doxil+ICI, sonopermeation+Doxil+ICI and ketotifen+sonopermeation+Doxil+ICI and were treated with ketotifen daily. When tumors reached an average size of 300 mm^3^, they were subjected to sonopermeation and one hour later Doxil and ICI were administered. Treatment with sonopermeation and nano-immunotherapy was repeated after four days. Primary tumors were removed and stored for further analysis two days after completion of the treatment protocol.

**Tumor stiffness and perfusion monitoring**. The elastic modulus and perfusion of tumors were monitored with ultrasound shear wave elastography (SWE) and contrast-enhanced ultrasound (CEUS), respectively. SWE was employed on a Philips EPIQ Elite Ultrasound system using a linear array transducer (eL18-4), according to previous research [55,56]. The method generates a shear wave velocity via an acoustic push pulse, creating a color mapped elastogram (in kPa) where red indicates hard and blue soft tissue. A confidence display is also used as a reference of the shear wave quality of the user-defined region of interest (ROI). The average value of the tumor region is automatically generated by the system under default scanner settings and expressed in kPa. The settings that were used were: frequency 10 MHz, power 52 %, B-mode gain 22 dB, dynamic range 62 dB. SWE was performed at two different planes of each tumor and the average value of both planes was used for our analysis.

CEUS was employed to assess tumor associated vascular perfusion after bolus injection of contrast agents (SonoVue 8 μl of sulphur hexafluoride microbubbles encapsulated by a phospholipid shell with a mean diameter of 2.5 μm, retro-orbital administration). Ultrasound scanning of tumors was performed using the linear array transducer L12-5. Contrast first harmonic signals were received at 8 MHz with a mechanical index of 0.06. For all subjects, the depth of the focus was set to 3 cm allowing measurements of the full depth of the tumor. Gain was set at 90 % for each recording. Focus was optimized and standardized for each subject when finding the tumor area using B-mode imaging. Real-time power modulation imaging was initiated after flashing imaging with a high mechanical index to destroy the microbubbles in tumor tissue to peak contrast intensity to allow visualization of bubble replenishment. Image analysis was performed offline using an ultrasound quantification and analysis software (QLAB, Phillips Medical Systems). Prior to each ultrasound application, mice were anesthetized by i.p. injection of Avertin (200 mg/kg) and ultrasound gel was applied to the imaging region to prevent any pressure of the transducer on the underlying tissue.

## Results

### Sonopermeation improves tumor perfusion and drug delivery in fibrosarcoma tumors

**Fig. 1:**
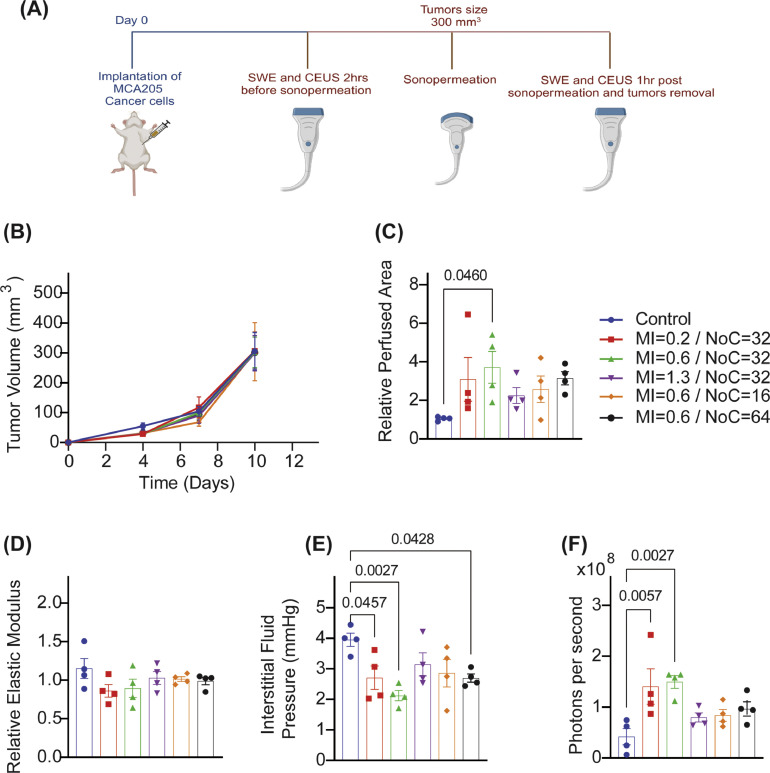
**Sonopermeation improves tumor perfusion and drug delivery in a parameter dependent manner.** (A) Experimental treatment protocol. Created with BioRender.com. (B) Tumor growth of MCA205 tumors (n = 8 mice). (C) Relative Perfused Area measured with CEUS and (D) Relative elastic Modulus measured with SWE, evaluated as the ratio of the measurements of Perfused Area or Elastic modulus 1 h post sonopermeation to the corresponding measurements 2hrs prior sonopermeation (n = 4 mice). (E) Interstitial fluid pressure (IFP) measured with the wick-in-needle technique (n = 4 mice). (F) Quantification of signal of fluorescence agents from *ex-vivo* imaging of tumors at 6h (n = 4 mice). Data are presented as mean ± SE. Statistical analyses were performed by using for (B) mixed-effects analysis with multiple comparisons Tukey test and for (C-F) ordinary one-way ANOVA with multiple comparisons Dunnett test.

First, we investigated the proper values of mechanical index (MI) and number of cycles (NoC) (based on the range values used previously [50], [51], [52], [53], [54]) to optimally improve the ability of sonopermeation to modulate tissue stiffness and increase perfusion and drug delivery *in vivo* in the MCA205 sarcoma model. When tumors reached an averaged size of 300mm^3^, we performed Shear Wave Elastography (SWE) and Contrast Enhanced Ultrasound (CEUS) to quantify tumor stiffness and perfusion 2hrs prior to sonopermeation [55,56] (Fig. 1A, B). Then we performed sonopermeation and studied its effect in tumor perfusion and elastic modulus with CEUS and SWE, respectively (Fig. 1C, D). Only the combination of MI=0.6 and NoC=32 increased with a statistically significant manner tumor perfusion 1hr after sonopermeation, while none of the combinations of the parameters affected the elastic modulus. Interstitial fluid pressure (IFP) was measured prior to tumor removal with the wick-in-needle method (Fig. 1E) [23,24]. Interestingly, all the combinations of sonopermeation parameters decreased IFP but the largest effect was observed with the combination of MI=0.6 and NoC=32. Then we checked the ability of various sonopermeation parameters to increase drug delivery in the MCA205 fibrosarcoma tumors. Specifically, 1hr after sonopermeation, we injected to the mice fluorescent DiR-micelles and 6hrs later we monitored *ex-vivo* their delivery in tumors (**Supplementary Fig. 2**). The largest effect was observed by the combination of MI=0.6 and NoC=32, enhancing drug delivery significantly compared to untreated mice (Fig. 1F). Based on the results, the optimal combination of parameters of sonopermeation was determined to be: MI=0.6 and NoC=32, and this was employed for the combination of sonopermeation with ketotifen.

### Ketotifen and sonopermeation optimally modulate the TME to enhance nano-immunotherapy

Next, we set out to investigate the main hypothesis of this research that the combination of the mechanotherapeutic ketotifen with sonopermeation can optimally improve perfusion and efficacy of nano-immunotherapy. In previous research, we found that 10mg/kg ketotifen, had a dual role acting both as a mechano-modulator to alleviate stiffness and mechanical forces in tumors and immuno-modulator by stabilizing mast cells within the TME of murine sarcoma models [31]. When MCA205 fibrosarcoma tumors reached 100 mm^3^, they were treated with ketotifen (10 mg/kg, i.p.) for three days and then subjected to sonopermeation. One hour later they received an intravenous (i.v.) injection of DiR-labelled micelles. Both monotherapies resulted in an increased accumulation of DiR-labelled micelles in the tumor site (**Supplementary Fig. 3**). However, the combination of ketotifen and sonopermeation further enhanced accumulation of fluorescent particles to tumors confirming that these treatments can synergistically improve delivery of nano-sized drugs.

**Fig. 2:**
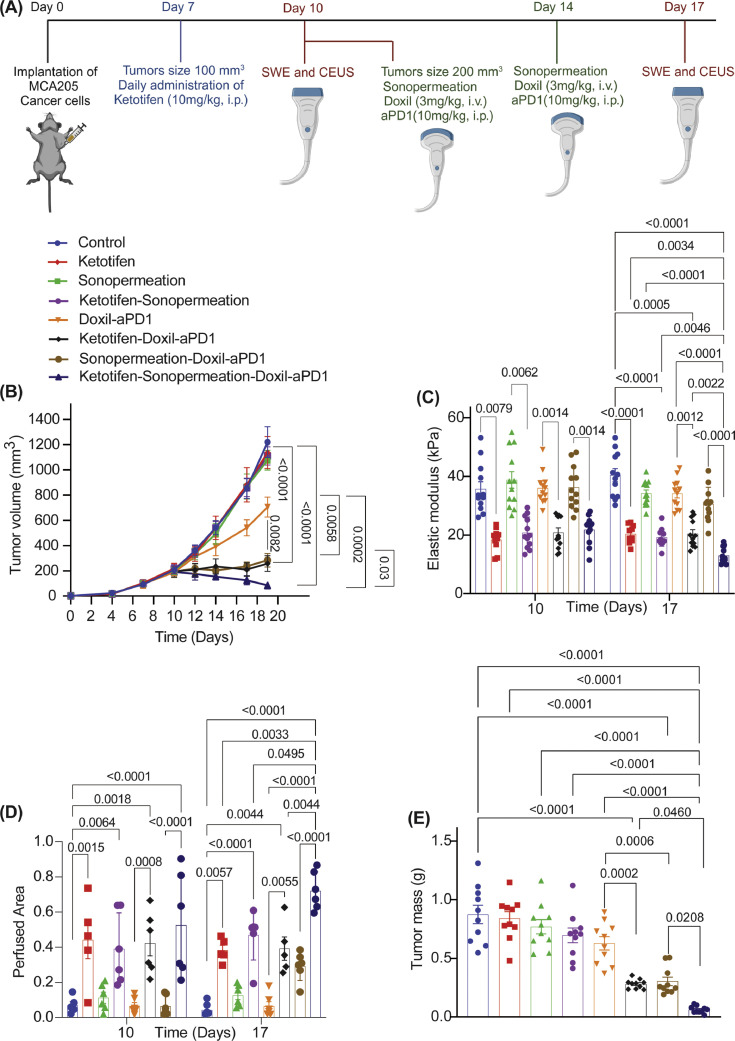
**Combination of ketotifen and sonopermeation significantly improve the efficacy of nano-immunotherapy in MCA205 fibrosarcoma tumors.** (A) Experimental treatment protocol. Created with BioRender.com. (B) Tumor growth (n = 10 mice), (C) Tumor Elastic modulus (n = 6 mice, N = 2 images field per mouse) measured with SWE, (D) Perfused area measured with CEUS (n = 6 mice), (E) tumor mass (n = 10 mice) of MCA205 tumors treated with Ketotifen, Sonopermeation, Doxil and anti-PD1 (aPD1). Statistical analyses were performed by using for (B) mixed-effects analysis with multiple comparisons Tukey test, for (C, D) using two-way ANOVA with multiple comparisons Dunnett test and for (E) ordinary one-way ANOVA with multiple comparisons Dunnett test.

**Fig. 3:**
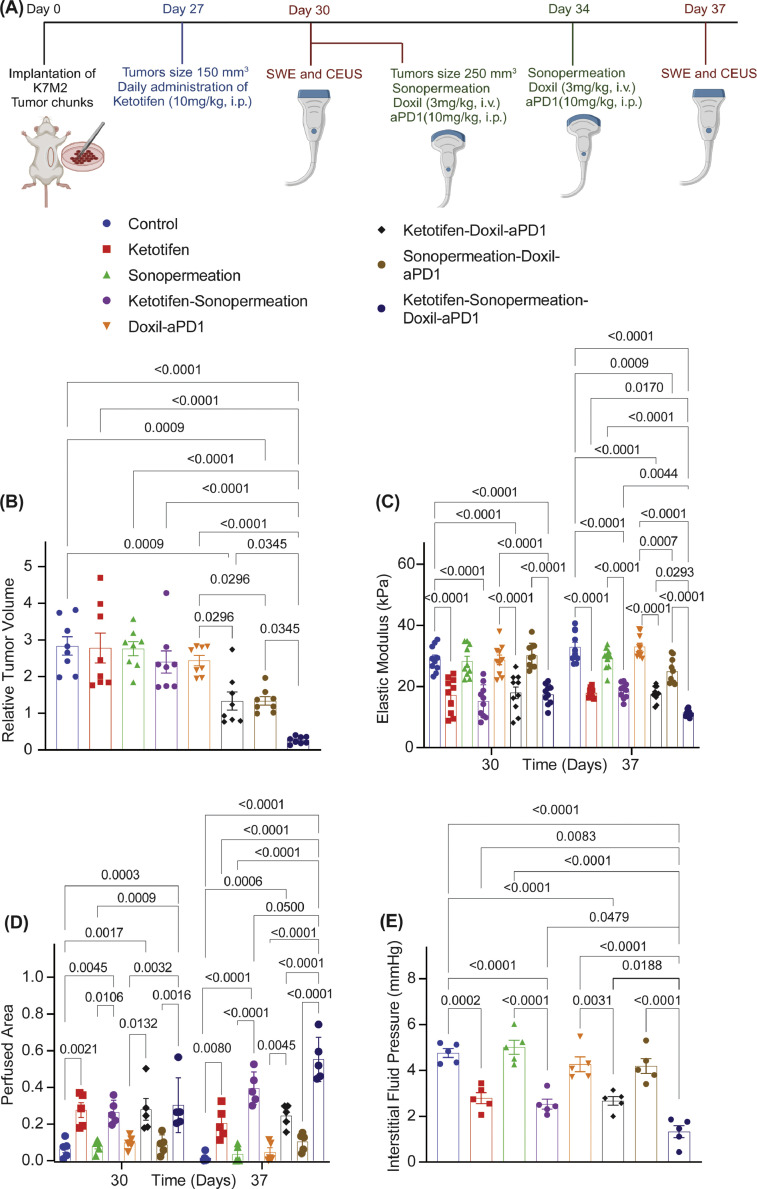
**Ketotifen and sonopermeation modulate tumor microenvironment and enhance efficacy of nano-immunotherapy in K7M2 osteosarcoma tumors.** (A) Experimental treatment protocol. Created with BioRender.com. (B) Relative tumor volume between Day 30 and Day 37 (n = 8 mice), (C) Tumor Elastic modulus (n = 5 mice, N = 2 images field per mouse) measured with SWE, (D) Perfused area measured with CEUS (n = 5 mice), (E) Interstitial fluid pressure (IFP) measured with the wick-in-needle technique (n = 5 mice) of K7M2 tumors treated with ketotifen, sonopermeation, Doxil and anti-PD-1 (aPD1). Statistical analyses were performed by using for ((B, E) ordinary one-way ANOVA with multiple comparisons Dunnett test and for (C, D) using two-way ANOVA with multiple comparisons Dunnett test and.

**Fig. 4:**
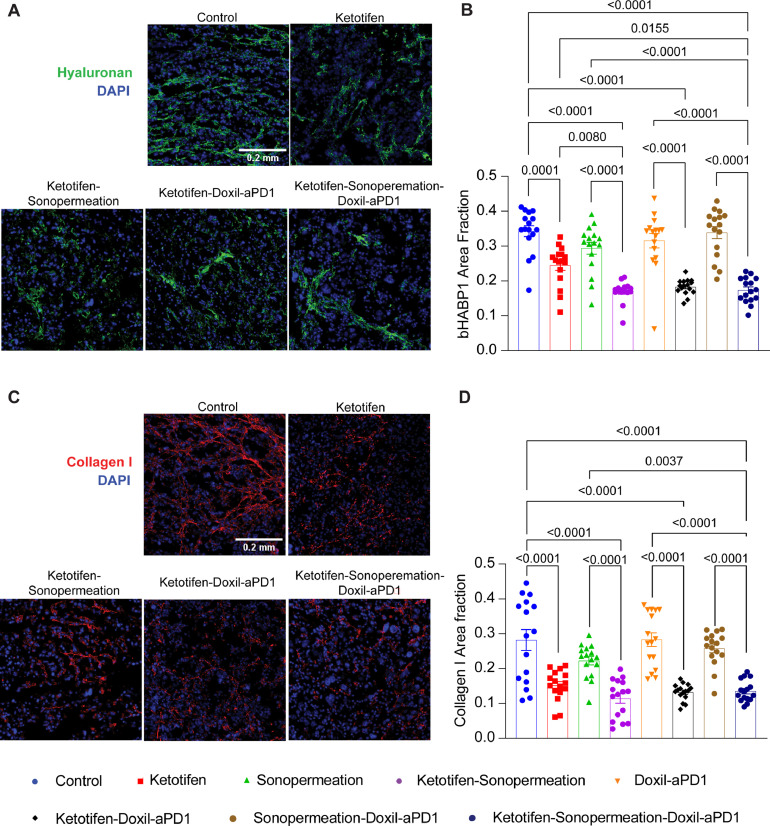
**Combination of ketotifen and sonopermeation with nano-immunotherapy decreased hyaluronan and collagen levels.** (A) Representative immunofluorescence images of hyaluronan binding protein (bHABP1, green) counterstained with nuclear staining (DAPI, blue) of K7M2 osteosarcoma tumors treated as indicated. White scale bar indicates 0.2 mm. (B) Graph of the area fraction of hyaluronan binding protein (bHABP1) in immunofluorescence images (n = 4 mice, N = 4 image fields). (C) Representative immunofluorescence images of Collagen I staining (red color) counterstained with nuclear staining (DAPI, blue) of K7M2 osteosarcoma tumors treated as indicated. White scale bar indicates 0.2 mm. (D) Graph of the area fraction of Collagen I in immunofluorescence images (n = 4 mice, N = 4 image fields). Data are presented as mean ± SE. Statistical analyses were performed by using ordinary one-way ANOVA with multiple comparisons Dunnett test.

Then, we investigated the ability of ketotifen and sonopermeation to enhance the antitumor efficacy of Doxil nanomedicine (size of 100nm) and an immune checkpoint inhibitor-ICI (size of 12nm). Specifically, MCA205 fibrosarcoma tumors and K7M2 osteosarcoma tumors were treated with control solution (anti-IgG, PBS), ketotifen (10 mg/kg, i.p.), sonopermeation (MI=0.6 and NoC=32) and Doxil (3mg/kg, i.v.) + ICI (anti-PD1, 10 mg/kg, i.p.) and their combinations (Figs. 2A, 3A). Monotherapies alone or the combination of sonopermeation with ketotifen had no antitumor effects in terms of reduction in tumor volume and mass (Figs. 2B, E, 3B, **Supplementary Fig. 4)**. However, combination of Doxil and anti-PD1 either with ketotifen or sonopermeation significantly improved antitumor efficacy of treatment. Importantly, the combination of ketotifen and sonopermeation with nano-immunotherapy optimally enhanced the therapeutic outcome. On the other hand, none of the treatments showed any toxicity effects to the mice (**Supplementary Fig. 5, Supplementary Fig. 6**). Tumors subjected to sonopermeation and ketotifen treatment prior to nano-immunotherapy, exhibited a statistically significant decrease in the tumor elastic properties measured with SWE, increased tumor perfusion measured with CEUS and decreased IFP compared to the group that received only ketotifen or ketotifen and nano-immunotherapy (Figs. 3C, D, 4C–E, S**upplementary Fig. 4, Supplementary Fig. 7, Supplementary Fig. 8).** This observation supports the argument that sonopermeation can enhance the TME modulation effects of the mechanotherapeutic ketotifen and to optimize therapeutic outcome (Fig. 2E). However, without treatment with ketotifen, sonopermeation alone or in combination with nano-immunothreapy showed moderate decrease in tissue stiffness and tumor perfusion. These results highlight that ketotifen and sonopermeation can have multiplicative synergistic effects on modulating the mechanical forces and improve perfusion in highly desmoplastic sarcomas and thus, optimize the efficacy of nano-immunotherapy.

The restoration of physical characteristics in desmoplastic tumors, as seen in the tumor models we investigated, is largely dependent on the levels of hyaluronan and collagen [4]. Ketotifen is an agent that targets these components [31] but the effect of sonopermeation is unknown. We assessed the levels of collagen and hyaluronan by immunofluorescence staining of tumor sections. Indeed, we found a decrease in collagen and hyaluronan protein levels in all groups received ketotifen (Fig. 4**, Supplementary Fig. 9)**. Importantly, the addition of sonopermeation and nano-immunotherapy in ketotifen further decreased hyaluronan levels supporting the previous results that combined therapy further decreased mechanical properties and improved tumor perfusion. Additionally, in the tumors subjected to sonopermeation without pre-treatment with ketotifen the decrease in collagen and hyaluronan was moderate in the same way with the decrease in tissue stiffness and perfused area of tumors.

**Fig. 5:**
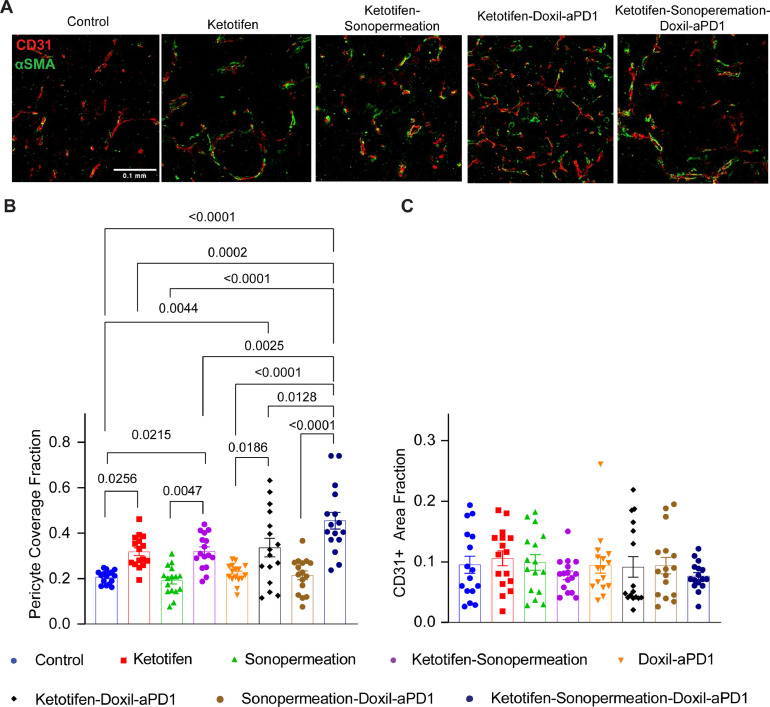
**Normalization of tumor blood vessels with ketotifen, sonopermeation and nano-immunotherapy.** (A) Representative immunofluorescence images of CD31 endothelial marker (red) and αSMA pericyte marker (green) of K7M2 osteosarcoma tumors treated as indicated. White scale bar indicates 0.1 mm. (B) Quantification of pericyte coverage fraction determined by the co-localization of CD31 and αSMA (n = 4 mice, N = 4 image fields). (C) Quantification of CD31 area fraction following immunostaining with anti-CD31 endothelial cell marker (n = 4 mice, N = 4 image fields). Data are presented as mean ± SE. Statistical analyses were performed by using ordinary one-way ANOVA with multiple comparisons Dunnett test.

Finally, we checked the impact of the combinatorial treatment on blood vessel pericyte coverage (Fig. 5, **Supplementary Fig. 10)**, which is a measure of vascular normalization. In tumors, as opposed to the host tissue, the lack of pericyte coverage results in heightened vessel permeability and a reduction in drug delivery. The use of ketotifen significantly increased the pericyte coverage in K7M2 tumors (Fig. 5B) whereas the total area of vessels remained unaffected (Fig. 5C). Furthermore, the addition of sonopermeation and nano-immunotherapy to ketotifen strengthen its capability to induce vascular normalization as the increase in pericyte coverage was statistically significant compared to all other groups treated with ketotifen.

### Combination of ketotifen and sonopermeation with nano-immunotherapy enhances T cells infiltration

**Fig. 6:**
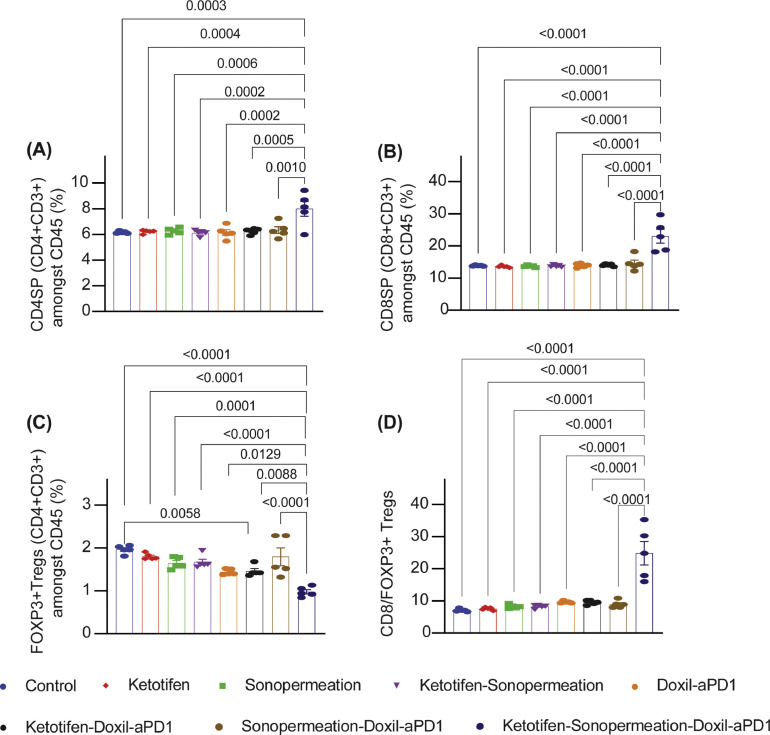
**Nano-immunotherapy combined with sonopermeation and ketotifen enhanced immune cells infiltration and decreased regulatory T cells**. Quantification of (A) CD4+ CD3+ (SP, single positive) and (B) CD8+ CD3+ (SP, single positive) cells amongst CD45+ lymphocytes and (C) Tregs defined as Foxp3+CD127loCD25hi CD4 SP gated on CD45+ lymphocytes (n = 5 mice). (D) Ratio of cytotoxic CD8+ T cells to immunosuppressive regulatory T cells (Tregs) (n = 5 mice). Data are presented as mean ± SE. Statistical analyses were performed by using ordinary one-way ANOVA with multiple comparisons Dunnett test.

Considering that tumor perfusion is associated with improved immune cell infiltration and activity, our objective was to investigate whether the robust antitumor effects of ketotifen and sonopermeation in combination with nano-immunotherapy was a result of enhanced tumor immunogenicity. Specifically, using flow cytometry analysis, we measured the T cells levels for the various treatments (**Supplementary Fig. 11).** Flow cytometry analysis revealed that only the combined treatment increased CD4^+^ T-helper cells and cytotoxic CD8^+^ T cells (Fig. 6A, B). Furthermore, only this treatment decreased immunosuppressive regulatory T cell (Tregs) and increased the ratio of cytotoxic CD8^+^ T cells to Tregs (Fig. 6C, D).

## Discussion

The failure of standard therapies to cure highly desmoplastic tumors is attributed in large part to insufficient and heterogeneous drug delivery to the tumor site owing to abnormalities in the TME that induce hypo-perfusion and hypoxia [57], [58], [59], [60]. Specifically, tumor stiffening and mechanical forces accumulated within solid components of tumors result in vessel compression [2,7,61]. A therapeutic strategy to decompress vessels and improve perfusion is the use of mechanotherapeutics to alleviate stiffness and mechanical forces in tumors [22]. The successful clinical translation of this strategy with the use of the common anti-hypertensive drug losartan as a TME normalization agent to improve therapeutic outcomes in locally advanced pancreatic cancer patients [30] highlights the promise of this new therapeutic strategy. However, preclinical data suggest that losartan and other normalization agents that we have tested have failed to fully restore blood vessel functionality and blood flow and almost half of the vessels remain compressed [23,24,26]. Ultrasound-mediated drug delivery with microbubbles is another strategy that could noninvasively enhance perfusion and thus, the transport of therapeutic agents to targeted tumors via sonopermeation. Sonopermeation aims to form transient pores in cell membranes and open the intercellular junctions of the endothelial cells that form the vessels facilitating a better transport for medicines in tumor tissues and allowing for selective and effective uptake by cancer cells. Furthermore, it has been recently reported that sonopermeation can reduce intratumoral solid stress and thus, improve delivery of nanomedicine [48], but the underlying mechanism is still unknown. It is clear, however, that in highly desmoplastic tumors with abundant collapsed vessels, microbubbles will not be able to effectively and uniformly delivered to the tumor leading to failure or compromised efficacy of this therapeutic strategy.

Here we have shown for the first time, that mechano-modulation of the tumor microenvironment by combining mechanotherapeutics and sonopermeation can have multiplicative synergistic effects on improving perfusion and therapeutic outcome. We employed ketotifen that we have shown in a previous study to have a dual role acting both as a mechano-modulator and immuno-modulator of the TME and it is being tested in clinical trials to improve chemotherapy in patients with sarcoma (EudraCT Number: 2022-002311-39) [31]. Here, we extended our previous study and we checked the efficacy of ketotifen with nano-immunotherapy. Furthermore, we employed a clinical ultrasound device to perform sonopermeation to identify the optimal parameters that can be used by clinicians to improve drug delivery using ultrasound with microbubbles. We demonstrated in murine sarcoma models that the combination of mechanotherapeutics with sonopermeation, compared to monotherapies alone, further enhanced tumor perfusion and decreased tissue elastic properties measured with ultrasound non-invasive techniques, such as SWE and CEUS. Comparing these results to our previous studies [19,23,24,[27], [28], [29]] where we have shown that mechanotherapeutics alone improved perfusion for about twice as much and reduced elastic modulus in half, in this study the combination of mechanotherapeutics with sonopermeation improved perfusion for more than 4 times and reduced the elastic modules of tumors for more almost 70% compared to the untreated tumors. Additionally, the combined therapy modulated TME by targeting extracellular matrix components and enhanced effector immune cells infiltration. Restoration of blood vessel functionality improved the antitumor efficacy of nano-immunotherapy and in conjuction with immunostimulation of the TME led to improved therapeutic outcomes.

In this study, we employed two sarcoma cell lines with different growth rates, the one with a very fast growth rate (the fibrosarcoma MCA205) and the other one with a lower growth rate (the osteosarcoma K7M2). This highlights that combination of ketotifen and sonopermeation can optimize the efficacy of nano-immunotherapy in murine models with different growth rates. However, testing the combined therapy in other models of cancer, such as transgenic or PDX models, could further confirm their efficacy to reprogram the TME and assist its clinical translation. Furthermore, the ability of the combined treatment to inhibit cancer metastasis in highly metastatic tumors and prolong overall survival should be investigated.

Our results highlighted that these two therapeutic strategies can be uniquely combined to overcome their limitations and thus, optimize the efficacy of nano-immunotherapy. The enhanced distribution of microbubbles in tumors owing to ketotifen treatment, improved the efficacy of sonopermeation. Additionally, sonopermeation complemented ketotifen to open up more tumor vessels, allowing more microbubbles entering the tumor and thus, creating a positive feedback loop. Due to the synergistic mechanisms of action and complementarity of ketotifen and sonopermeation, the two strategies should be combined to have multiplicative effects on improving perfusion and therapeutic outcome in clinical studies. All methods and agents used in this study are non-invasive and clinically approved, which makes the results highly transferable to the clinic.

## Data availability statement

Data can be retrieved from corresponding author upon reasonable request.

## CRediT authorship contribution statement

**Fotios Mpekris:** Writing – review & editing, Writing – original draft, Visualization, Validation, Supervision, Resources, Project administration, Methodology, Investigation, Funding acquisition, Formal analysis, Data curation, Conceptualization. **Myrofora Panagi:** Writing – review & editing, Validation, Methodology, Investigation, Formal analysis. **Antonia Charalambous:** Writing – review & editing, Validation, Methodology, Investigation, Formal analysis. **Chrysovalantis Voutouri:** Writing – review & editing, Validation, Methodology, Investigation, Formal analysis. **Christina Michael:** Methodology, Writing – review & editing, Validation, Investigation, Formal analysis. **Antonia Papoui:** Writing – review & editing, Validation, Methodology, Investigation, Formal analysis. **Triantafyllos Stylianopoulos:** Writing – review & editing, Visualization, Validation, Supervision, Resources, Project administration, Methodology, Investigation, Funding acquisition, Formal analysis, Data curation, Conceptualization.

## Declaration of competing interest

The authors declare that the research was conducted in the absence of any commercial or financial relationships that could be construed as a potential conflict of interest.

## Appendix Supplementary materials

Image, application 1
