# Supplementary material for: A synergistic approach for modulating the tumor microenvironment to enhance nano-immunotherapy in sarcomas
Source: Neoplasia. 2024 Mar 22;51:100990. doi: 10.1016/j.neo.2024.100990 (PMC10978543; doi:10.1016/j.neo.2024.100990)
Supplement: Supplementary file 1 [file mmc1.docx]

**Supplementary Material**

**A synergistic approach for modulating the tumor microenvironment to enhance nano-immunotherapy in sarcomas**

Fotios Mpekris, Myrofora Panagi, Antonia Charalambous, Chrysovalantis Voutouri, Christina Michael, Antonia Papoui, Triantafyllos Stylianopoulos

**Accumulation of fluorescence micelles in murine sarcoma tumors.** Sonopermeation response study. C57BL/6 female mice bearing orthotopic MCA205 tumors of 300 mm^3^ were randomized into six groups (n=4 mice per group) as follows: one control group, and five groups subjected to sonopermeation with the following values of parameters: 1) Mechanical Index=0.2 and Number of Cycles=32, 2) Mechanical Index=0.6 and Number of Cycles=32, 3) Mechanical Index=1.3 and Number of Cycles=32, 4) Mechanical Index=0.6 and Number of Cycles=16 and 5) Mechanical Index=0.6 and Number of Cycles=64. Mice were subjected to sonopermeation treatment as described in main manuscript and 1hr later an i.v. injection of DiR-labelled micelles (96nm diameter) was administered. Six hours post injection of micelles mice were sacrificed and primary tumors were removed scanned at 20% excitation power for 5 sec at 745 nm excitation and 870 nm emission wavelength using the AMI-HT imaging system.

Ketotifen and Sonopermeation response study. C57BL/6 female mice bearing orthotopic MCA205 tumors of 100 mm^3^ were randomized into four groups (n=4 mice per group): 1) Control group, 2) Ketotifen (10 mg/kg, i.p.), 3) Sonopermeation and 4) Ketotifen+Sonopermeation. Mice were treated with Ketotifen daily for three days. Then mice were subjected to sonopermeation and an i.v. injection of DiR-labelled micelles (96nm diameter) was administered. Six hours post injection of micelles mice were sacrificed and primary tumors were removed scanned at 20% excitation power for 5 sec at 745 nm excitation and 870 nm emission wavelength using the AMI-HT imaging system.

**Preparation of fluorescent micelles.** Fluorescent micelles for drug delivery studies were prepared according to our previous study [1]. Specifically, the fluorescence imaging agent 1,1’-dioctadecyltetramethyl indotricarbocyanine iodide (DiR) was loaded into the [BzMA_36_-*co*-DEAEMA_6_]-*b*-HEGMA_89_ diblock copolymer micelles. Initially, the diblock copolymer [BzMA_36_-*co*-DEAEMA_6_]-*b*-HEGMA_89_ (MW = 31685 g mol^-1^, 37.5 mg, 0.0012 mmol) was placed in a glass vial (50 mL) with a screw cap and it was left to dissolve in acetone (12 mL) under stirring conditions at room temperature. The resulting diblock copolymer solution was then centrifuged at 2000 g for 10 min. Continuously, DiR (3 mL from stock solution prepared in acetone, solution concentration: 0.25 g L^-1^) was transferred into the diblock copolymer solution, followed by the addition of PBS (15 mL). The resulting mixture was left to stir overnight in dark conditions at room temperature allowing the acetone to evaporate. The aqueous micellar solution was then filtered through 0.45 μm cellulose acetate filter. Unbound DiR was removed by ultrafiltration in PBS using a dialysis tube Slide-A-Lyzer with a molecular cut-off of 2 kDa. The DiR loading concentration was determined by recording the UV-vis spectrum of the micellar solution at 750 nm (characteristic absorption wavelength of DiR) using the corresponding calibration curve.

**Interstitial fluid pressure.** Interstitial fluid pressure (IFP) was measured *in vivo* using the previously described wick-in-needle technique after mice were anesthetized with i.p. injection of Avertin and prior to tumor excision [2].

**Fluorescence immunohistochemistry.** Tumors were excised, washed in 1x PBS twice for 10 min and incubated in 4 % PFA at 4 °C for 24 h. The samples were washed twice in 1x PBS for 10 min, dehydrated in successive steps of alcohol and xylene, and finally embedded in paraffin. Serial sections (7 μm) of paraffin-embedded tissues were produced using the microtome (Accu-Cut SRM 200 Rotary Microtome, SAKURA). The sections were flattened out into water and allowed to dry overnight at 37 °C, deparaffinized, and rehydrated.

**Hyaluronan binding protein and collagen staining and quantification.** Antigen retrieval was performed on tissue sections (microwave heat treatment with TriSodium Citrate, pH 6, for 20 min), washed with 1x PBS/ 0.025 % Tween20, and incubated in blocking solution (15 % FBS, 1,5 % BSA, 0.3 % Tween20) for 2 h at room temperature (RT). Afterwards, slides for hyaluronan staining were incubated with biotinylated hyaluronan binding protein (b-HABP, AMS.HKD-BC41, amsbio 1:100) overnight at 4 °C. Next, the sections were incubated with streptavidin-FITC conjugate (SA1001, Invitrogen 1:1000) and DAPI nuclear stain at RT for 2 h in the dark. Slides for collagen staining were incubated with Collagen antibody (ab21286, Abcam 1:100) overnight at 4 °C. Next, the sections were incubated with Alexa Fluor-488 anti-rabbit IgG (H+L) (A11034, Invitrogen 1:400) and DAPI nuclear stain at RT for 2 h in the dark. For all stained tissue sections, confocal z-stacks were acquired with 1 μm z-spacing on a Leica STELLARIS microscope using a 20x objective. The entire tissue section was imaged using the spiral-scan function of LAS X Navigator. Quantification of the area fraction of hyaluronan was quantified as the number of pixels of immunofluorescence image with signal intensity above a threshold determined using a control slide without primary antibody normalized to the number of pixels positive for DAPI stain. The images were analyzed using custom and built-in algorithms in MATLAB (MathWorks, Inc., Natick, MA, USA).

**Pericyte coverage staining and quantification.** Pericytes were identified with αSMA/CD31 colocalization. Briefly, tissue cryosections were cut to 10 μm and blocked (10 % FBS, 3 % donkey serum, 0.3 % Tween-20 in 1x PBS) overnight at 4 °C. αSMA was double-stained with CD31. The tissue sections were incubated with rabbit anti-αSMA (ab5694, Abcam 1:100) and rat anti-CD31 (553370, BD Pharmingen, 1:50) overnight at 4 °C. After washing, tissue sections were then incubated with Alexa Fluor-488 anti-rabbit IgG (H+L) (A11034, Invitrogen 1:400) and -647 anti-rat IgG (H+L) (A21247, Invitrogen 1:600) secondary antibodies and DAPI for 1 h at RT. The areas positive for pericyte marker (αSMA) and endothelial cell marker (CD31) were quantified separately as pixels of the immunofluorescence image with signal intensity above a threshold determined using a control slide without primary antibody. Then, pericyte coverage fraction was determined using the number of pixels with colocalized signal (αSMA^+^ and CD31^+^) divided by the total number of CD31^+^ pixels.

**Flow cytometry.** On day 19 of treatment, MCA205 fibrosarcoma tumors (n=10 per treatment group) were harvested in cold RPMI, minced into fine fragments and incubated with 1 ml reconstituted Liberase (0.5 mg per tumor, Roche) for 30 min at 37 ^o^C on an end-over-end shaker. Enzymatic digestion was ceased by the addition of RPMI media containing 10% FBS and 1% antibiotic/antimycotic solution. The resulting tissue homogenates were filtered through 40 μm cell strainers and single cell suspensions were collected and counted. 1x10^6^ cells per sample were then incubated with fixable viability dye (Invitrogen) for gating of viable cells. Non-specific antibody binding was blocked following incubation with the rat anti-mouse CD16/CD32 mAb (BD Bioscience) for 10min at RT. Cells were labeled with the various fluorochrome conjugated antibodies, washed and resuspended in 0.5% BSA, 1x PBS buffer. The anti-mouse antibodies used in the experiment are the following; CD4-PerCP-Cy5.5 (GK1.5, BioLegend), CD8a-V450 (53-6.7, eBioscience), Foxp3-FITC (FJK-16s, Invitrogen), CD45-V500 (30-F11, BD Bioscience), CD25-PE-Cy7 (PC61.5, BD Bioscience), CD3-PE (145-2C11, BD Bioscience), CD11b-V450 (M1/70, eBioscience), F4/80-APC (BM8, BioLegend), CD206-PE-Cy7 (C068C2, BioLegend), CD127-APC (A7R34, BioLegend), Gr-1-PE (RB6-8C5, BioLegend), CD38-PerCP-Cy5.5 (90, BioLegend). Flow cytometry data were obtained using BD FACSLyric III flow cytometer and analyzed using BD FACS Suite software. Data presented is representative of singlets, live cells.

**Supplementary Table 1.** Treatment groups for sonopermeation response study

| **Number of Group** | **Treatment** |
| --- | --- |
| 1 | Control |
| 2 | Mechanical Index=0.2  Number of Cycles=32 |
| 3 | Mechanical Index=0.6  Number of Cycles=32 |
| 4 | Mechanical Index=1.3  Number of Cycles=32 |
| 5 | Mechanical Index=0.6  Number of Cycles=16 |
| 6 | Mechanical Index=0.6  Number of Cycles=64 |

**Supplementary Figures**

**
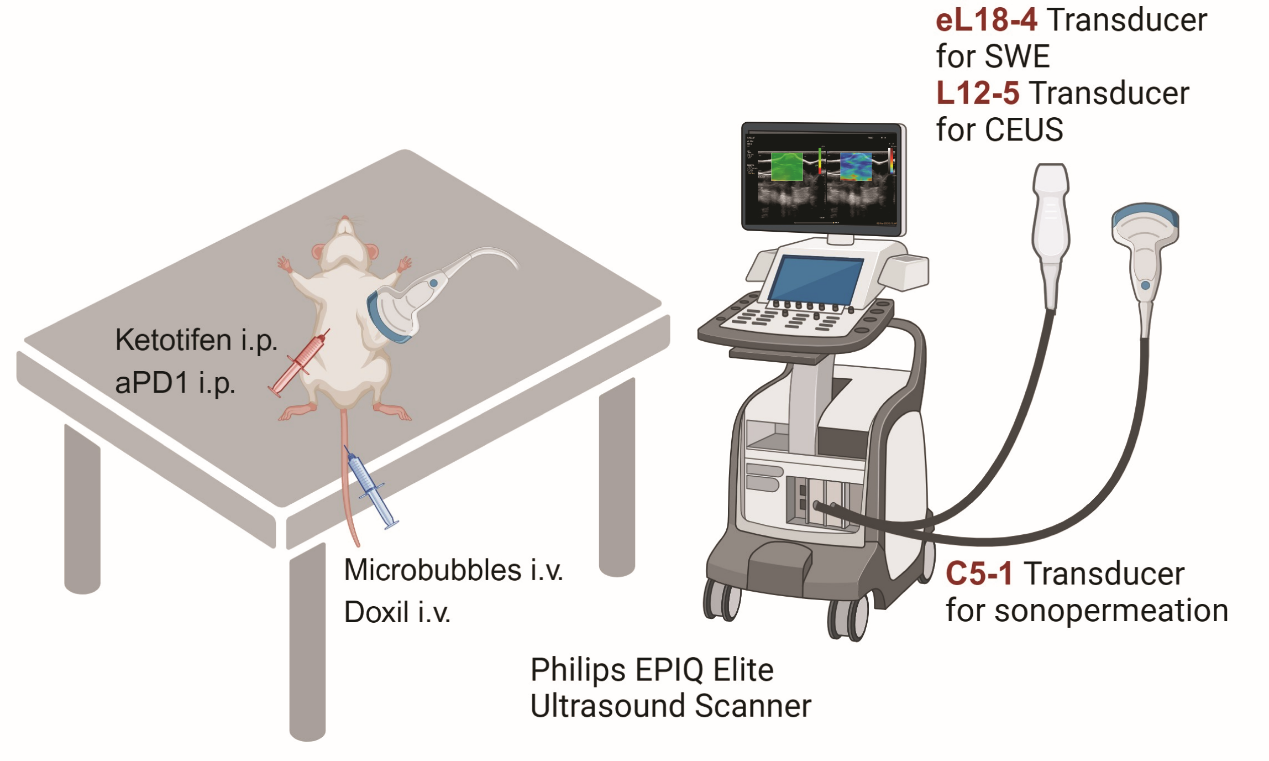
**

**Supplementary Fig. 1*.* Experimental setup for mice treatment.**

**
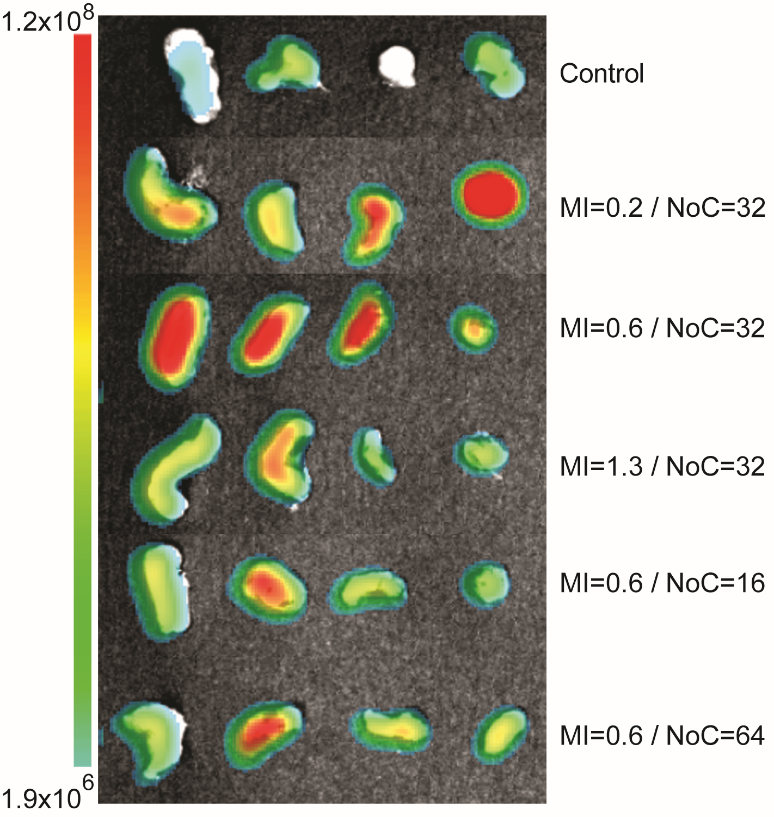
**

**Supplementary Fig. 2*.* Sonopermeation improves drug delivery in MCA205 tumors.** Ex-vivo imaging of DiR-micelles distribution in tumors, 6hrs post administration, using different parameters of Mechanical Index (MI) and Number of Cycles (NoC). Micelles were administered 1hr after sonopermeation.

**
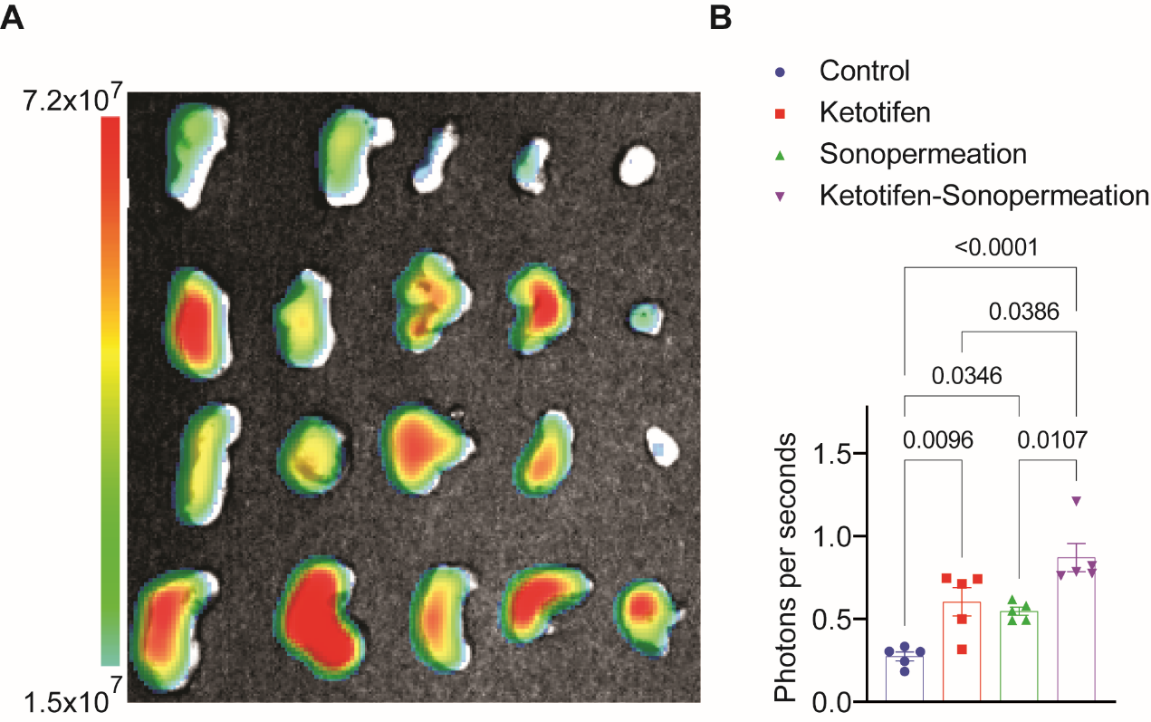
**

**Supplementary Fig. 3. Ketotifen and sonopermeation increase drug delivery in fibrosarcoma tumors.** (A) Ex-vivo imaging of micelles distribution in tumors in mice (n=5) treated with ketotifen and/or sonopermeation after injection with fluorescent DiR-micelles. (B) Quantification of signal from ex-vivo imaging of tumors at 6hrs. Data are presented as mean ± SE. Statistical analyses were performed by using ordinary one-way ANOVA with multiple comparisons Dunnett test.


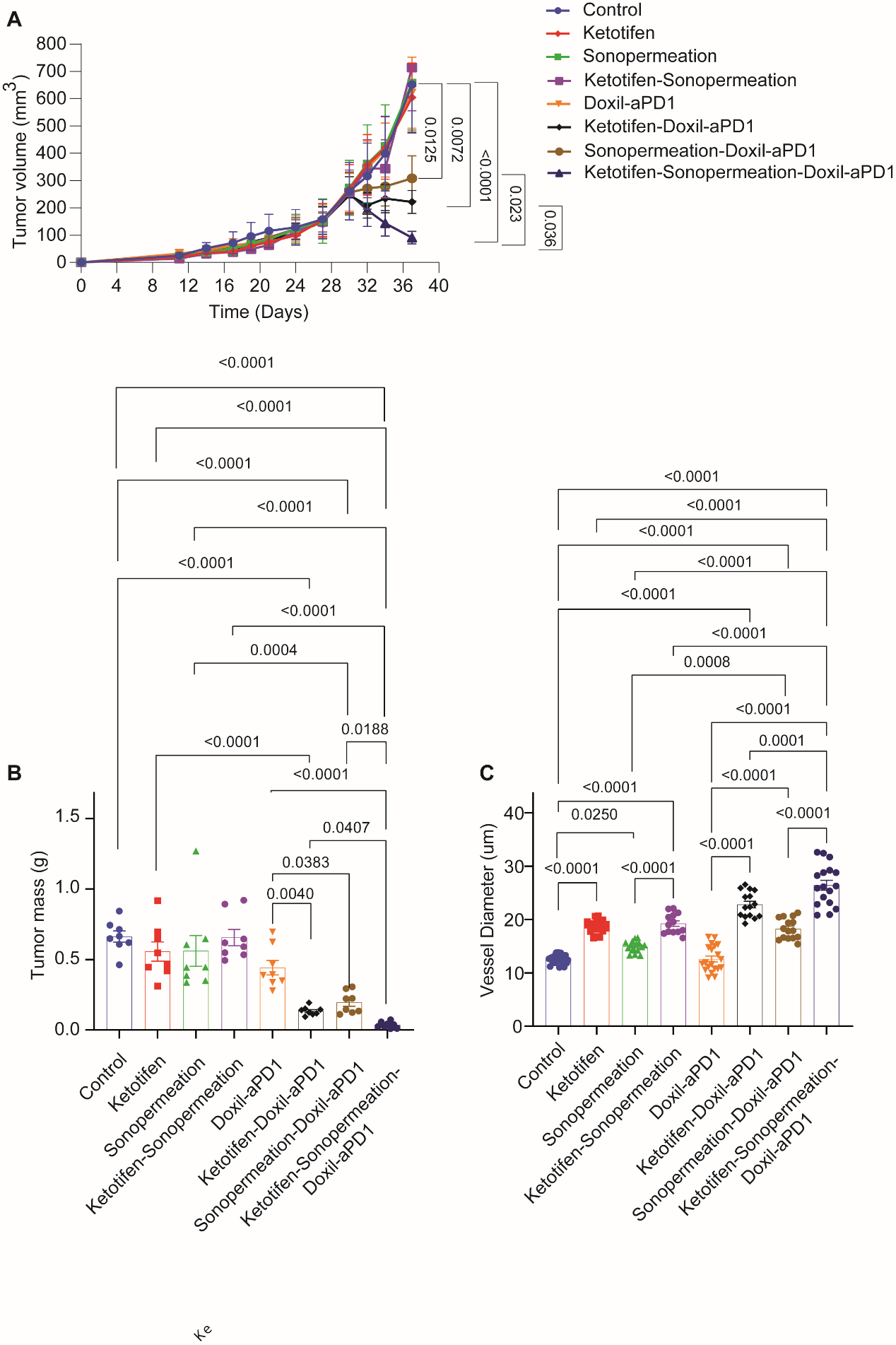


**Supplementary Fig. 4*.* Ketotifen and sonopermeation enhance treatment efficacy of nano-immunotherapy in K7M2 osteosarcoma tumors.** (A) Tumor growth (n = 8 mice), (B) tumor mass (n = 8 mice) and (C) vessel diameter of K7M2 tumors treated with Ketotifen, Sonopermeation, Doxil and anti-PD1 (aPD1). Data are presented as mean ± SE. Statistical analyses were performed by using for (A) mixed-effects analysis with multiple comparisons Tukey test and for (B, C) ordinary one-way ANOVA with multiple comparisons Dunnett test.

**
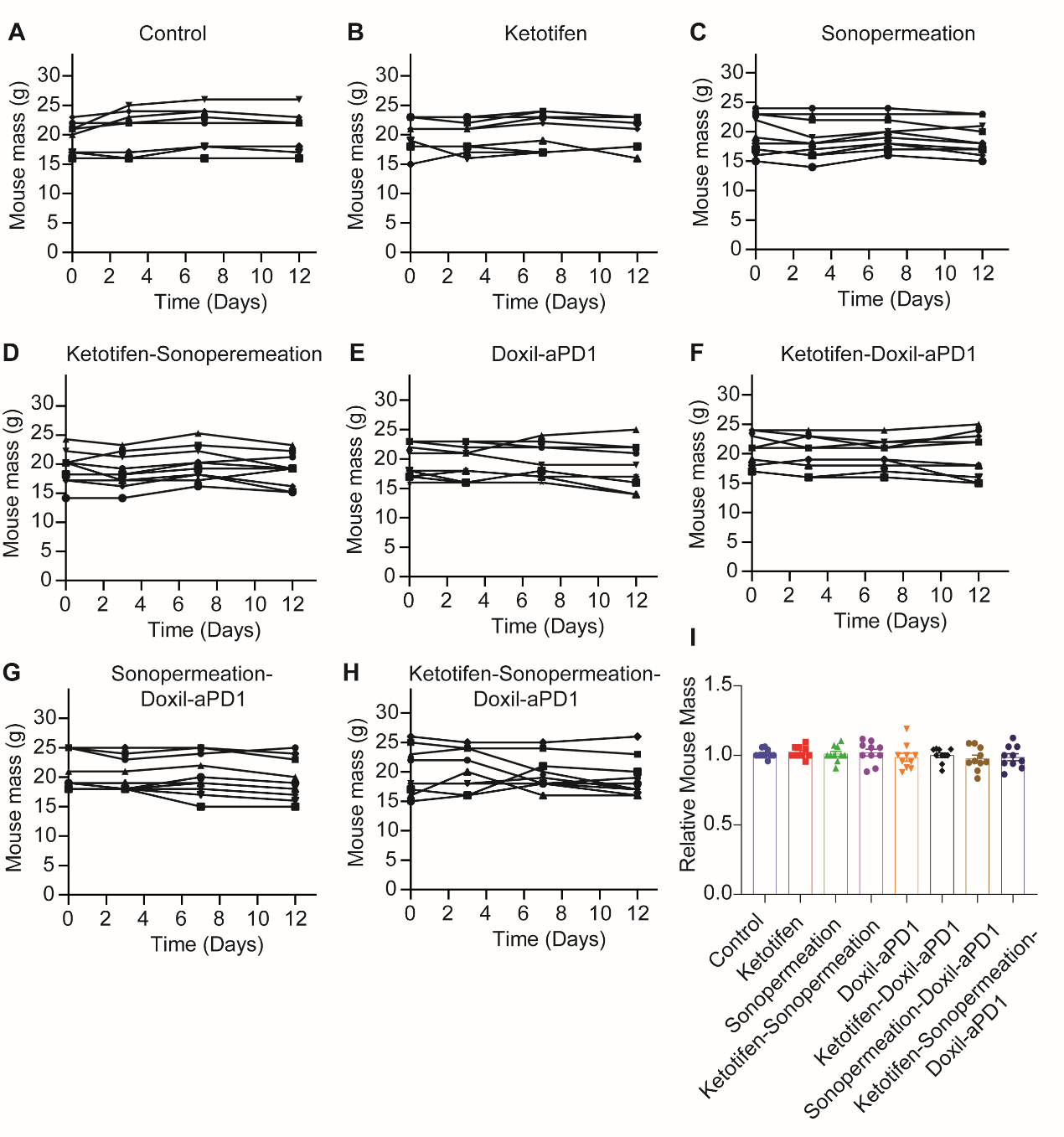
**

**Supplementary Fig. 5*.*** (A-H) Mouse weight over the treatment course per group after treatment with ketotifen (Day 0) for the MCA205 study. (I) Relative mouse mass between Day 0 (initiation of treatment with ketotifen) and Day 12 (tumor removal).


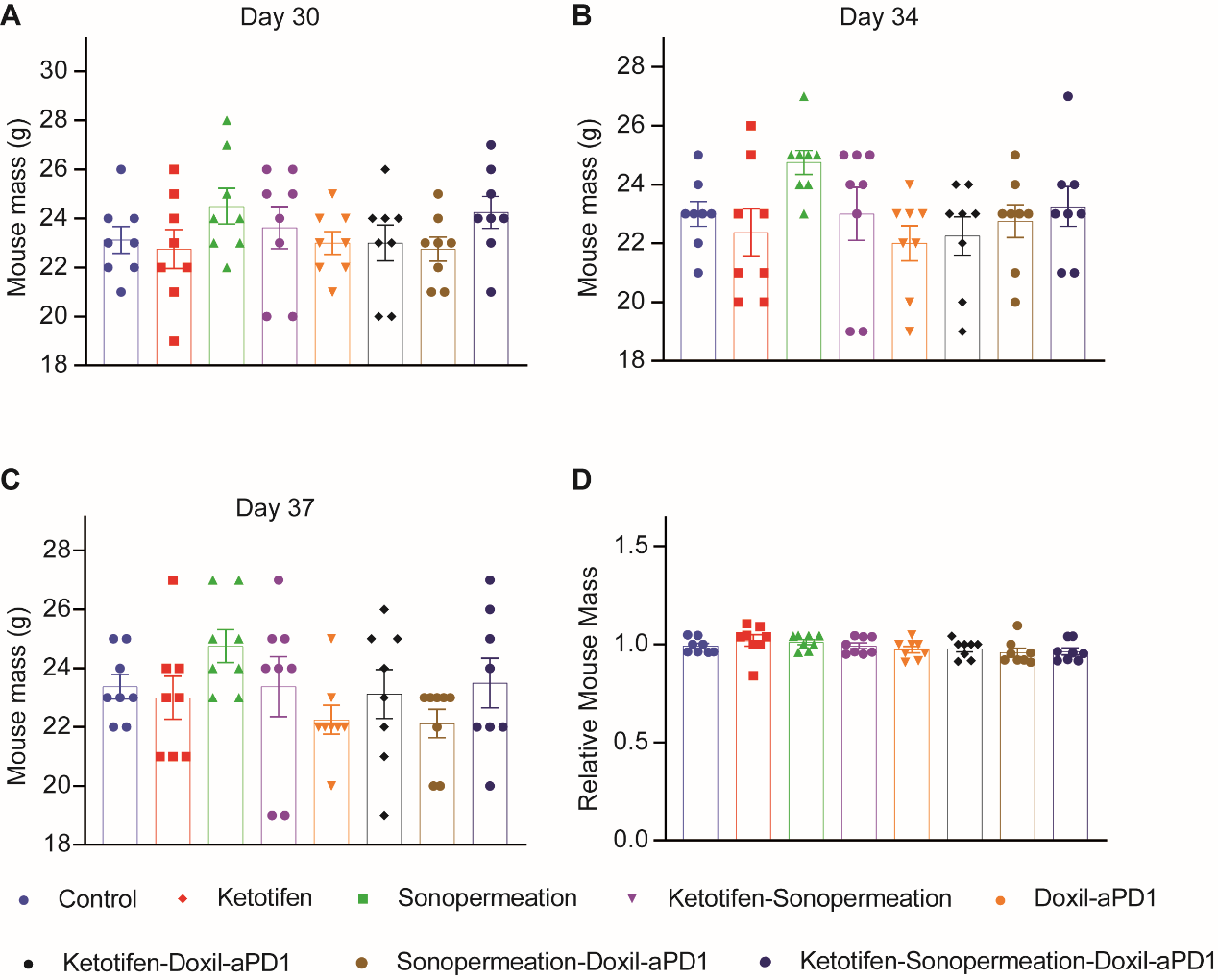


**Supplementary Fig. 6.** Mouse weight over the treatment course per group on Days (A) 30, (B) 34 and (C) 37 for the K7M2 tumors. (D) Relative mouse mass between Day 30 (initiation of treatment with sonopermeation and Doxil-aPD1) and Day 37 (tumors removal).

***
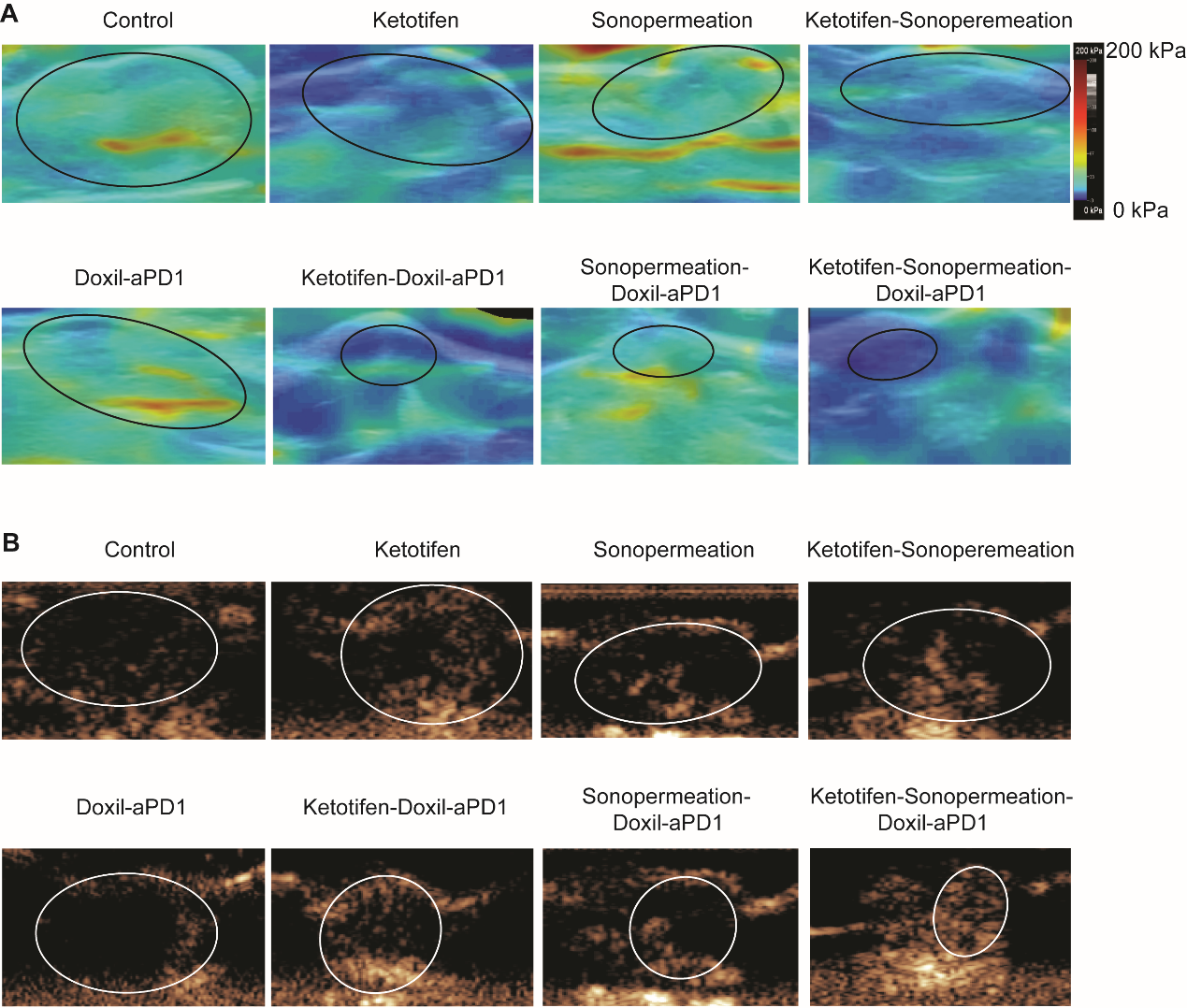
***

**Supplementary Fig. 7.** (A) Representative SWE images of MCA205 tumors for the various treatment groups at the end of the experimental protocol. The black line denotes the tumor margin. (B) Representative images of microbubbles’ spatial distribution with CEUS at the time of peak intensity and at the end of the experimental protocol for MCA205 tumors. The white line denotes the tumor margin.

**
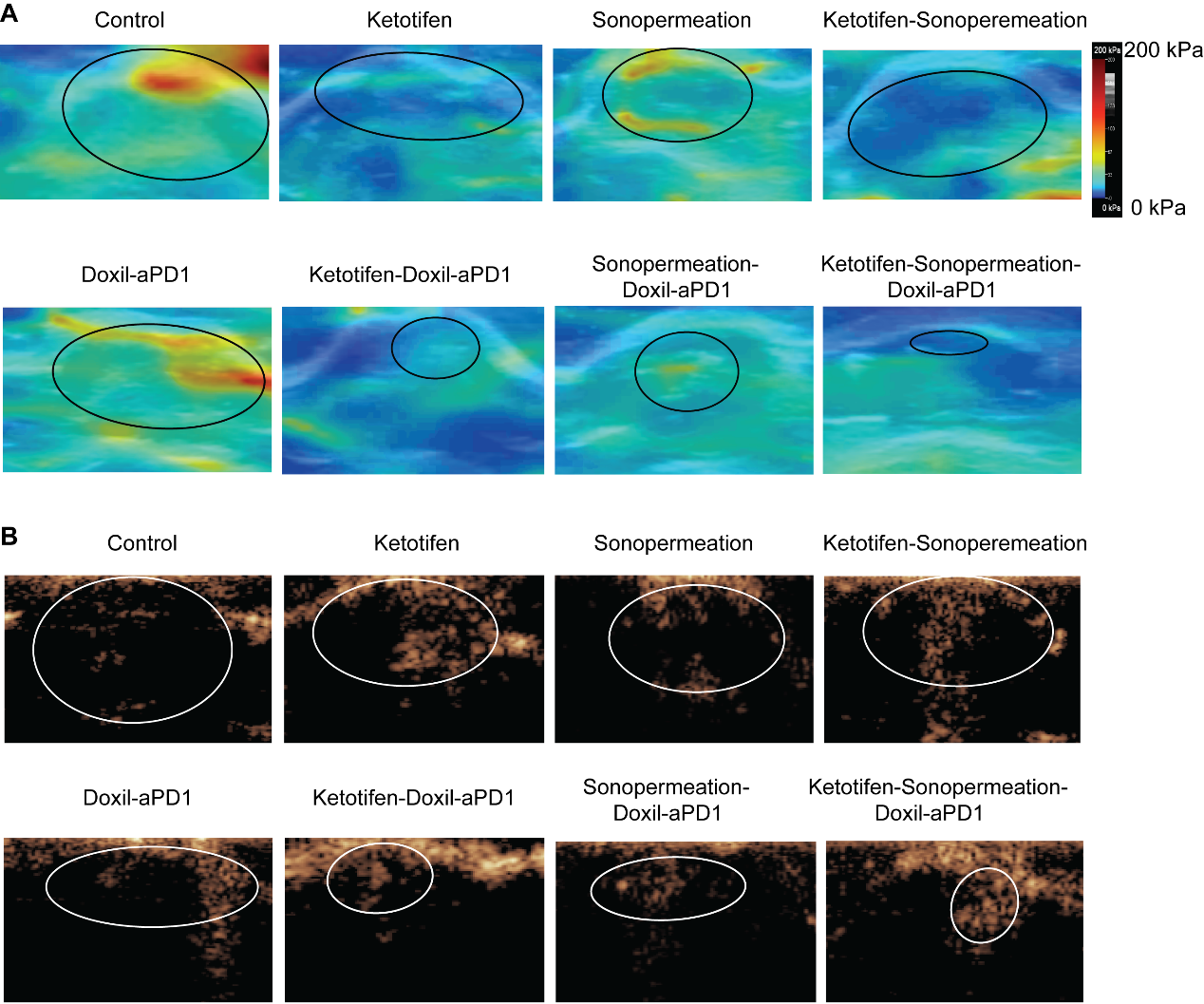
**

**Supplementary Fig. 8.** (A) Representative SWE images of K7M2 tumors for the various treatment groups at the end of the experimental protocol. The black line denotes the tumor margin. (B) Representative images of microbubbles’ spatial distribution with CEUS at the time of peak intensity and at the end of the experimental protocol for K7M2 tumors. The white line denotes the tumor margin.

**
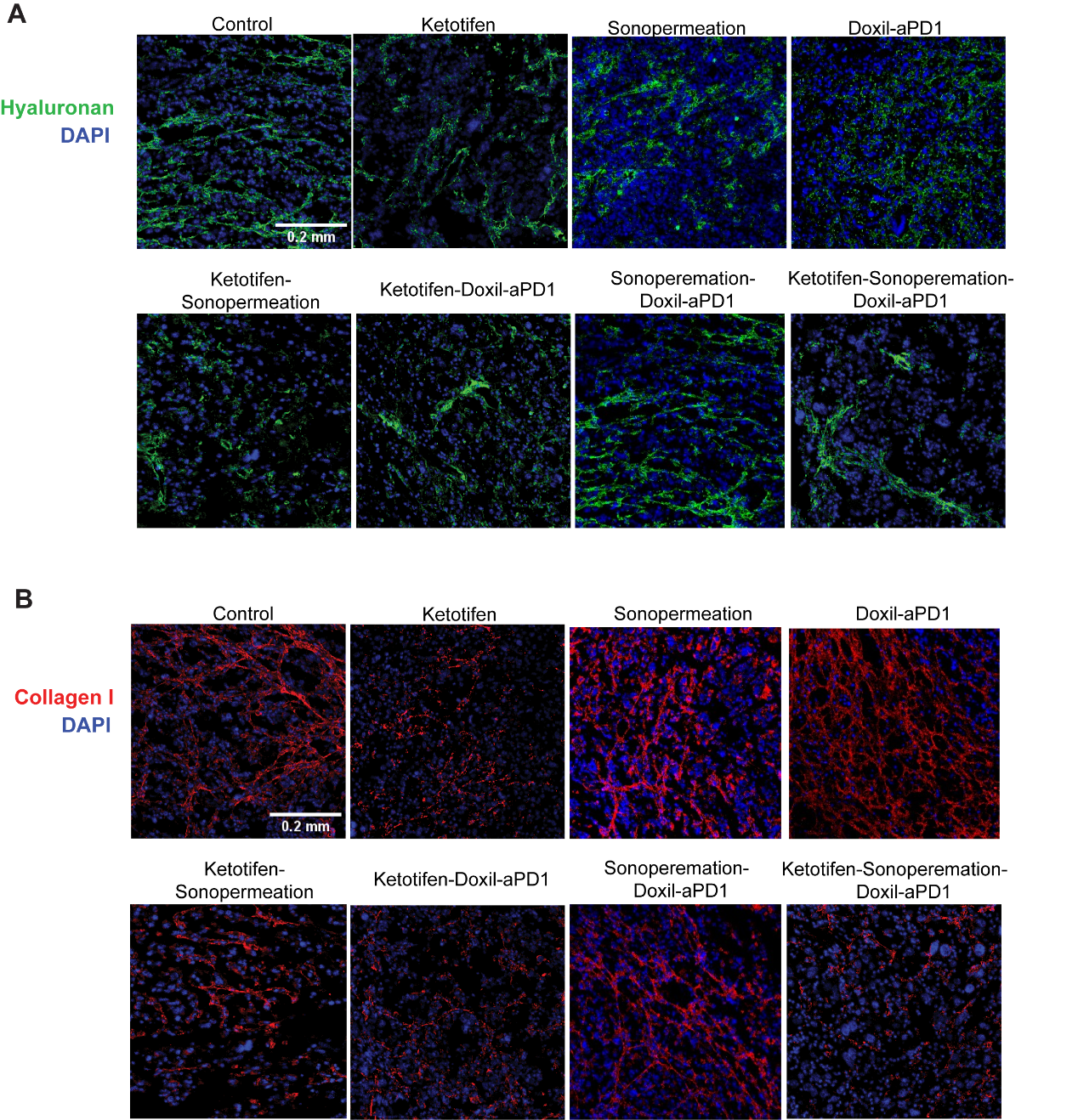
**

**Supplementary Fig. 9.** (A) Representative immunofluorescence images of hyaluronan binding protein (bHABP1, green) counterstained with nuclear staining (DAPI, blue) of K7M2 osteosarcoma tumors treated as indicated. White scale bar indicates 0.2 mm. (C) Representative immunofluorescence images of Collagen I staining (red color) counterstained with nuclear staining (DAPI, blue) of K7M2 osteosarcoma tumors treated as indicated. White scale bar indicates 0.2 mm.

**
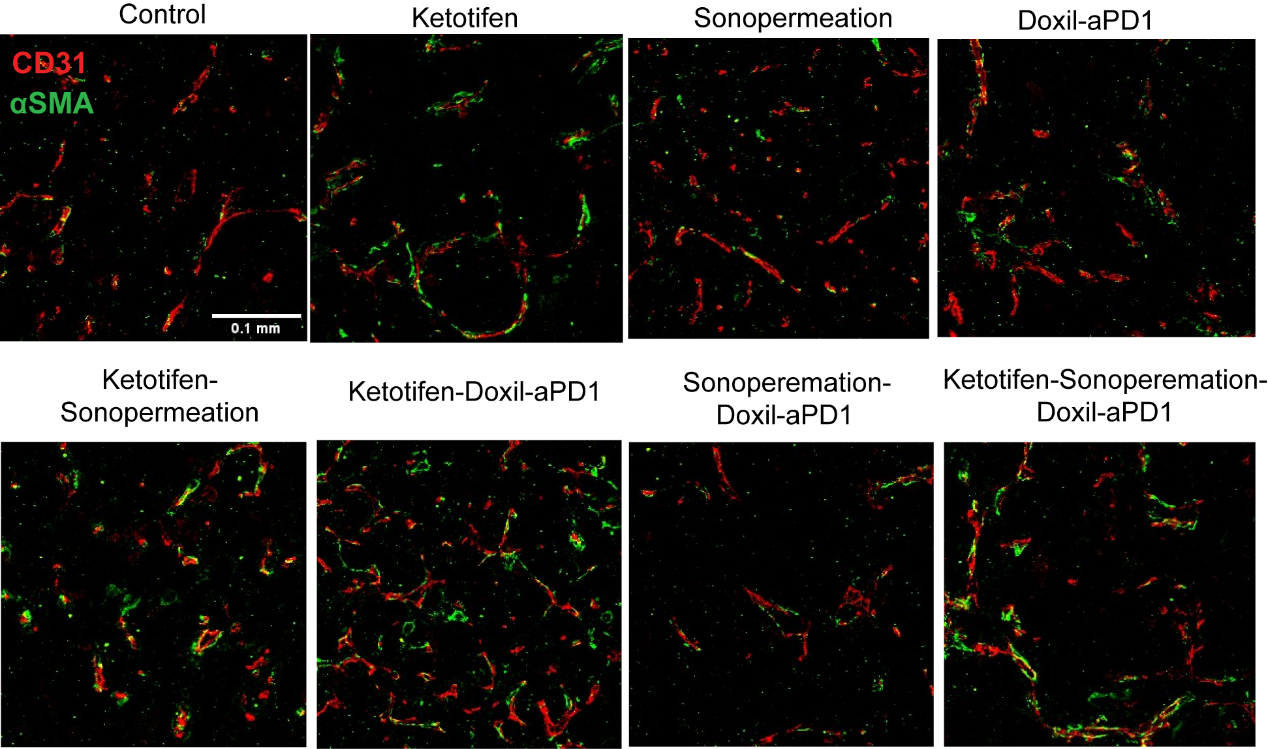
**

**Supplementary Fig. 10.** Representative immunofluorescence images of CD31 endothelial marker (red) and αSMA pericyte marker (green) of K7M2 osteosarcoma tumors treated as indicated. White scale bar indicates 0.1 mm.


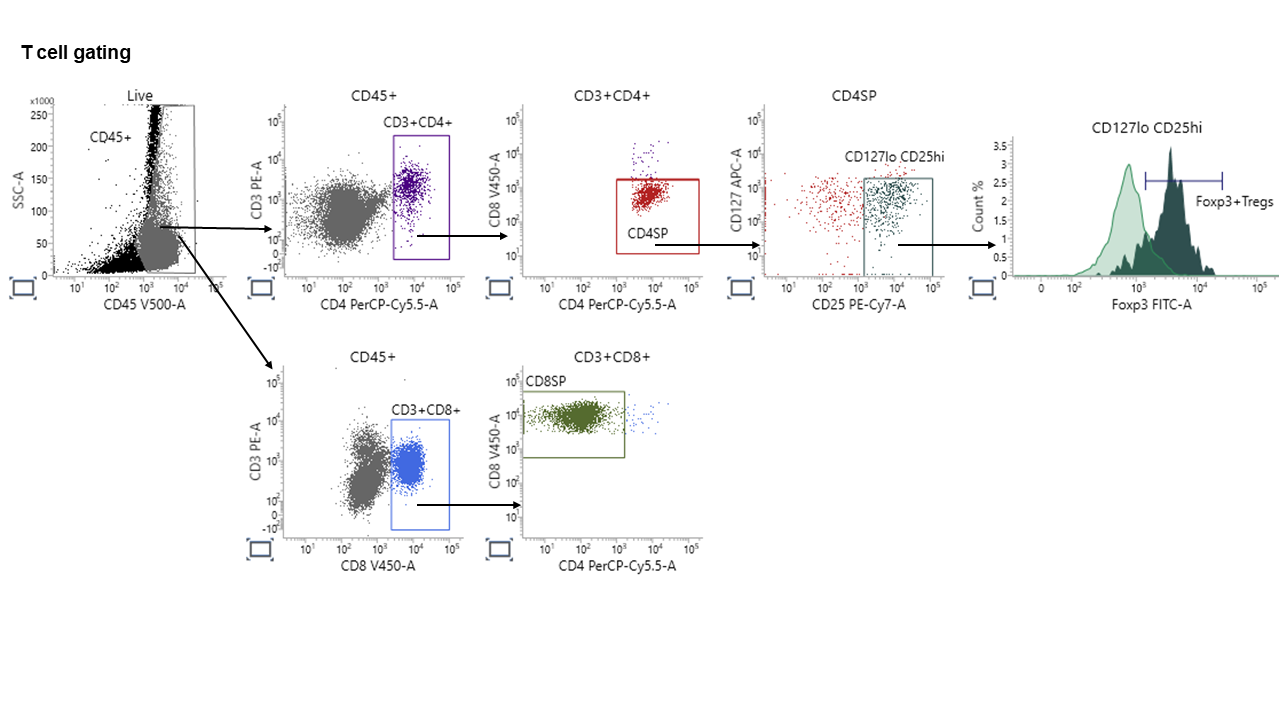


**Supplementary Fig. 11.** Gating strategy with representative histograms of flow data corresponding to T cell immunostaining.

**References**

[1] F. Mpekris, P.C. Papaphilippou, M. Panagi, C. Voutouri, C. Michael, A. Charalambous, M.M. Dinev, A. Katsioloudi, M. Prokopi-Demetriades, A. Anayiotos, H. Cabral, T. Krasia-Christoforou, T. Stylianopoulos (2023). Pirfenidone-loaded polymeric micelles as an effective mechanotherapeutic to potentiate immunotherapy in mouse breast cancer models. ACS Nano, 17, 24, 24654–24667.

[2] T. Stylianopoulos, J. D. Martin, V. P. Chauhan, S. R. Jain, B. Diop-Frimpong, N. Bardeesy, B. L. Smith, C. R. Ferrone, F. J. Hornicek, Y. Boucher, L. L. Munn, and R. K. Jain. (2015). Causes, consequences, and remedies for growth-induced solid stress in murine and human tumors. Proc. Natl. Acad. of Sci. 109, 15101-15108.
